# High Doses of Romiplostim Induce Proliferation and Reduce Proplatelet Formation by Human Megakaryocytes

**DOI:** 10.1371/journal.pone.0054723

**Published:** 2013-01-24

**Authors:** Manuela Currao, Carlo L. Balduini, Alessandra Balduini

**Affiliations:** 1 Dept. of Molecular Medicine, Biotechnology Laboratories, IRCCS Policlinico San Matteo Foundation and University of Pavia, Pavia, Italy; 2 Dept. of Internal Medicine, IRCCS Policlinico San Matteo Foundation and University of Pavia, Pavia, Italy; 3 Dept. of Biomedical Engineering, Tufts University, Medford, Massachusetts, United States of America; Naval Research Laboratory, United States of America

## Abstract

**Background:**

Romiplostim (AMG531) is a Thrombopoietin (TPO) receptor agonist with no homology with the endogenous TPO that has been used to treat patients affected by immune thrombocytopenia (ITP). Despite the use of TPO mimetics in the clinical practice, the mechanisms underlying their impact on megakaryocyte function is still unknown.

**Methodology/Principal Findings:**

In this project we took advantage of an *in vitro* human model, that we have established in our laboratory for long time to study megakaryocyte development from human cord blood-derived progenitor cells, and we demonstrated that increasing doses of AMG531 (100 to 2000 ng/mL) determine a progressive increase of megakaryocyte proliferation with a parallel decrease in megakaryocyte ploidy and capacity of extending proplatelets. Most importantly, these differences in megakaryocyte function seemed to be correlated to modulation of AKT phosphorylation.

**Conclusions/Significance:**

Overall our results shed new light on the mechanisms and on the relevance of dosage related to AMG531 impact on megakaryocyte function.

## Introduction

Megakaryopoiesis is a multiple stage differentiation process under the control of thrombopoietin (TPO) [1]. Megakaryocyte (MK) differentiation is marked by the development of progressive polyploidy and increase of protein production [2], followed by terminal differentiation leading to cytoskeletal reorganization, proplatelet formation and platelet release [3], [4]. Endogenous TPO supports the maturation of MKs and stimulates platelet production through activation of three major downstream signal transduction pathways: JAK-STAT (Janus kinase–signal transducers and activators of transcription), PI3K-AKT (phosphoinositol-3-kinase/AKT) and two MAPK (mitogen-activated protein kinase), ERK1/2 (extracellular signal-related kinase 1/2) and p38 [5]–[7]. Romiplostim (AMG531) is a peptide TPO receptor agonist that has no sequence homology with endogenous thrombopoietin and has been recently approved for treatment of certain patients with immune thrombocytopenia [8]–[9]. However, the mechanistic basis of AMG531–induced platelet production are not completely understood. Therefore, the purpose for the present work was to compare recombinant human TPO (rHuTPO) to AMG531 in terms of functional and mechanistic outcome in an *in vitro* human model of megakaryopoiesis.

## Materials and Methods

### Cell Culture

Human cord blood was collected following normal pregnancies and deliveries upon informed consent of the parents, in accordance with the ethical committee of the IRCCS Policlinico San Matteo Foundation and the principles of the Declaration of Helsinki. CD34^+^ cells from human cord blood samples were separated and cultured as previously described [10]. Briefly, CD34^+^ progenitor cells were separated using immunomagnetic beads selection (Miltenyi Biotec; Bergish Gladbach, Germany) and cultured, for 13 days, in Stem Span medium (Stem cell Technologies; Vancouver, Canada) supplemented with 1% L-glutamine, 100 U/ml penicillin, 100 ng/mL streptomicyn, 10 ng/mL IL-6, IL-11, 10 or 100 ng/mL rHuTPO (PeproTech EC Ltd, London, UK), and 100, 1000 or 2000 ng/mL AMG531 at 37°C in a 5% CO_2_ fully humified atmosphere. MO7e cell line (Genetics Institute, Boston, MA) was cultured in Dulbecco’s Modified Eagle’s Medium (DMEM) supplemented with 1% L-glutamine, 100 U/ml penicillin, 100 ng/mL streptomicyn, 10% Fetal Bovine Serum (FBS) and 50 ng/mL GM-CSF (PeproTech EC Ltd).

### Megakaryocyte Differentiation and Morphological Analysis

MK yield was evaluated at the end of culture as previously described [10]. Briefly cells were harvested, cytospun on glass coverslips, fixed with 4% paraformaldehyde (PFA) and stained with a primary antibody against CD61 (clone SZ21) (Immunotech, Marseille, France) to evaluate MK output. Nuclear counterstaining was performed with Hoechst 33258 (100 ng/ml in Phosphate Buffer Saline, PBS). Specimens were mounted in Pro Long Antifade Reagent (Invitrogen, Milan, Italy). Images were acquired by an Olympus BX51 microscope (Olympus, Deutschland GmbH, Hamburg, Germany) using a 63×/1.25 UPlanF1 oil-immersion objective. Negative controls were routinely performed by omitting the primary antibody. MKs were identified on the basis of CD61 expression.

### Proplatelet Formation

To analyze proplatelet formation by MKs, 12 mm glass coverslips were coated with 100 µg/mL fibrinogen (Sigma, Milan, Italy), for 2 hours at room temperature (RT) and subsequently blocked with 1% Bovine serum albumin (BSA) (Sigma, Milan, Italy) for 1 hour at RT. At day 13 of culture cells were harvested, plated onto substrate-coated coverslips in 24-wells plates, and allowed to adhere for 16 hours at 37**°**C and 5% CO_2_. After 16 hours cells were fixed in 4% PFA, permeabilized with 0.1% Triton X-100, and double-stained with anti-α-tubulin antibody (clone DM1A) (Sigma, Milan, Italy) and goat polyclonal CD61 antibody (St. Cruz Biotechnology, Heidelberg, Germany). Images acquired by Olympus BX51 microscope using a 20×/0.5 UPlanF1 objective or 63×/1.25 oil immersion objective. MKs extending proplatelets were identified as CD61^+^ cells extending α-tubulin positive long filamentous structures. Proplatelet formation was evaluated after 16 hours by fluorescence microscopy.

### Western Immunoblotting

In order to analyze biochemical pathways activated in MO7e megakaryoblastic cell line and human MKs by rHuTPO and AMG531, cells were starved for 16 hours in Iscove’s modified Dulbecco’s medium (IMDM, Gibco, Grand Island, NY, USA) and then stimulated for 10 minutes with different concentrations of rHuTPO or AMG531 before lysis. Thereafter, in order to analyze the effect of prolonged incubation with these growth factors, CD34^+^ cells were cultured for 13 days with rHuTPO or AMG531 before lysis. For western blot analysis MO7e or MKs were lysed as previously described [11]. Samples containing equal amounts of proteins were subjected to electrophoresis on 5–15% gradient polyacrylamide gel, transferred to polyvinylidine fluoride (PVDF) membranes and visualized using the enhanced chemiluminescent system. Primary antibodies and dilution are listed in Table 1.

**Table 1:** List of primary antibodies used in Western Blotting analysis.

Antigen	Type	Dilution	Manufacturer
pAKT	RbP	1∶1000	Cell Signaling
AKT	RbP	1∶1000	Cell Signaling
pERK 1/2	RbM	1∶1000	Millipore
ERK1/2	MM	1∶2000	Cell Signaling
pp38	RbP	1∶1000	Abcam
p38	MM	1∶1000	Abcam
CD61	GP	1∶500	Santa Cruz
RUNX-1	MM	1∶1000	Sigma
NFE2	RbP	1∶1000	Gene Tex
β-ACTIN	MM	1∶5000	Sigma

RbP, rabbit polyclonal; RbM, rabbit monoclonal; MM, mouse monoclonal; GP, goat polyclonal.

### Ploidy Analysis

For ploidy analysis cells were fixed in 70% cold ethanol at −20°C overnight, centrifuged at 1500×g for 10 minutes and then resuspended in PBS containing 1 µg/mL Propidium Iodide (Sigma) and 50 µg/mL RNAse (Sigma) and incubated with 10 µg/mL anti-human CD41 conjugated with FITC (eBioscience Inc, San Diego, USA) for 30 minutes at room temperature in the dark. Samples were analyzed on a Becton Dickinson FACSCalibur flow cytometer. Data were analyzed using Cell Quest analysis software.

### Statistics

Data are presented as mean ± SD. One-way ANOVA followed by the *post-hoc* Bonferroni’s *t*-test was used to analyze data, with a significant difference set at p<0.05. All experiments were independently performed at least 3 times.

## Results and Discussion

**Figure 1:**
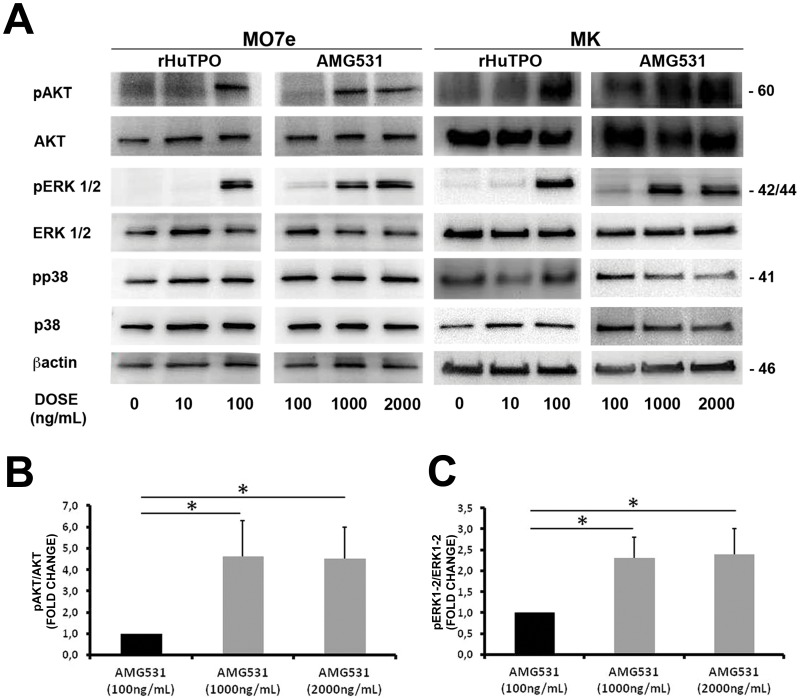
Biochemical pathways activated by rHuTPO and AMG531. Both megakaryoblastic cell line MO7e and mature MKs were deprived of growth factors for 16 hours and then stimulated with different concentrations of rHuTPO or AMG531. (A) Cells were lysed and pERK1/2, pAKT pp38 were detected by western blot analysis. β-actin was revealed to demonstrate equal protein loading. (B-C) Histograms show densitometric analysis of AKT (B) and ERK1/2 (C) phosphorylation levels in MO7e and MK stimulated with different concentration of AMG531. Results are presented as fold change of pAKT/AKT and pERK1-2/ERK1-2 ratios in cells cultured with AMG531 1000 and 2000 ng/mL with respect to cells cultured with AMG531 100 ng/mL. The error bars represent the mean ± SD of 3 independent experiments. *p<0.05.

**Figure 2:**
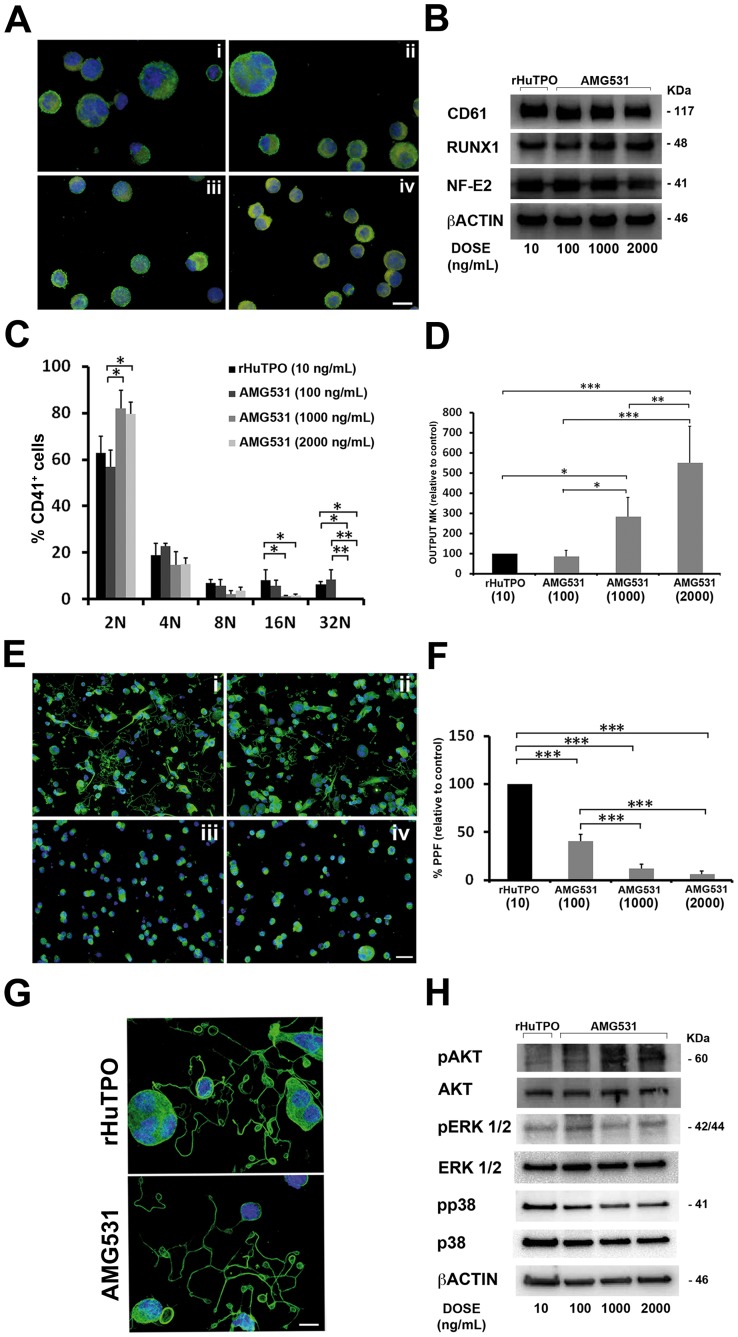
Effect of AMG531 on MK development. MKs were differentiated from human umbilical cord blood progenitors and cultured for 13 days in presence of AMG531 at different concentrations. 10 ng/mL of rHuTPO was used as control. (A) Immunofluorescence images of MKs cultured with rHuTPO 10 ng/mL (i) or AMG531 100 ng/mL (ii), 1000 ng/mL (iii) and 2000 ng/mL (iv) at day 13 of culture. Cell samples were citospun on 12 mm cover slips and stained with anti-CD61 antibody (green)**.** Nuclei were counterstained with Hoechst 33288 (blue). Images were acquired through an Olympus BX51, magnification 63×. Scale bar = 25 µm. (B) Western blot analysis of MK differentiation markers CD61, RUNX-1, NFE2. Samples were probed with anti β-actin antibody to demonstrate equal loading. (C) Ploidy levels of MKs was analyzed by flow cytometry as described in Design an Methods. Data are expressed as mean ± SD of 3 different experiments. *p<0.05, ** p<0.01. (D) MK output was calculated as the percentage of CD61^+^ cells at day 13 of culture and normalized to the total number of CD34^+^ cells at the beginning of the cell culture (values in parenthesis represent rHuTPO or AMG531 concentrations, expressed as ng/mL). The error bars represent the mean ± SD of 6 independent experiments. *p<0.05, ** p<0.01, ***p<0.001. (E) Immunofluorescence images of proplatelet formig-MKs derived from cultures treated with rHuTPO 10 ng/mL (i) or AMG531 100 ng/mL (ii), 1000 ng/mL (iii) and 2000 ng/mL (iv) stained with anti α-tubulin antibody (green). Nuclei were stained with Hoechst 33288 (blue). Images were acquired through an Olympus BX51, magnification 20×. Scale bar = 50 µm. (F) Histogram shows proplatelet formation (PPF) analyzed after 16 hour adhesion on fibrinogen (values in parenthesis represent rHuTPO or AMG531 concentrations, expressed as ng/mL). The error bars represent the mean ± SD of 5 independent experiments. ***p<0.001. (G) Representative images of proplatelet revealed with anti α-tubulin staining (green). Nuclei were counterstained with Hoechst 33288 (blue). Images were acquired through an Olympus BX51, magnification 63×. Scale bar = 20 µm. (H) Western blot analysis of pAKT, pERK1/2 and pp38 in MKs at day 13 of culture. AKT, ERK1/2, p38 and β-actin were revealed to show equal loading.

**Figure 3:**
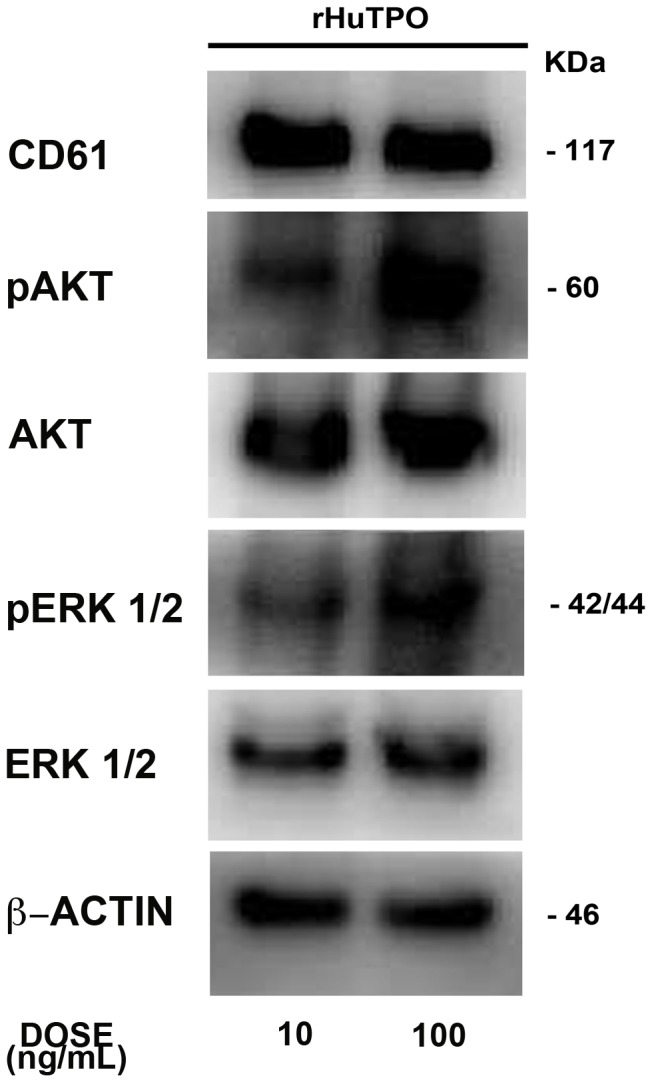
Effect of rHuTPO concentration on AKT and ERK1/2 phosphorylation. MKs were differentiated from human umbilical cord blood progenitors and cultured for 13 days in presence of rHuTPO at different concentrations. Western blot analysis of pAKT, pERK1/2 in MKs at day 13 of culture. AKT, ERK1/2 and β-actin were revealed to show equal loading.

AMG531 has been demonstrated to promote platelet production in immune thrombocytopenia (ITP) as well as hepatitis C patients [12]–[15]. Importantly, AMG531 was shown to bound to c-mpl and promote the growth of colony-forming units-megakaryocyte (CFU-Meg) in a concentration-dependent manner [16]. Endogenous TPO activates PI3K-AKT and MAPK downstream signaling pathways: AKT and ERK1/2 have been shown to play a crucial role in Mk maturation and platelet formation, while, at present, there is no functional evidence for a role of p38 MAPKs [11], [17]–[19]. Therefore, to learn more about how rHuTPO and AMG531 affect MK function, we first evaluated their effects on activation of ERK1/2, AKT and p38 in human MKs and MO7e cell line cultured in a serum/cytokine-free medium overnight and subsequently stimulated for 10 minutes with increasing concentrations of either rHuTPO or AMG531. rHuTPO and AMG531 concentrations were determined on the basis of our prior studies [20] and literature [8], [16]. As shown in **Figure 1A**, activation of ERK1/2 and AKT resulted concentration-dependent in both rHuTPO and AMG531 treated cells, while constant activation was observed for p38. Specifically, increased levels of pERK1/2 and pAKT were observed upon stimulation with 100 ng/mL rHuTPO and 1000 ng/mL AMG531 [8]. Importantly, 1000 ng/mL of AMG531 represented the concentration peak as higher concentrations did not determined additional increase of ERK1/2 and AKT phosphorylation levels (**Figure 1B–C**). On these basis, we further analyzed the impact of prolonged incubation with different concentrations of AMG531 in an *in vitro* system for human MK development. Human umbilical cord blood derived CD34^+^ cells were cultured in the presence of increasing doses of AMG531 and functional outcomes were compared in terms of MK differentiation and output, ploidy, proplatelet formation and morphology. Since no differences in MK differentiation or function were observed with increasing concentrations of rHuTPO (data not shown) we took 10 ng/mL of rHuTPO as the reference concentration for our experiments. After two weeks of culture all AMG531 doses, as well as rHuTPO, promoted the differentiation of most of the progenitors cells (75±5% for rHuTPO, 74±3%, 73±4%, 73±5% for AMG 100, 1000 and 2000 respectively) into MKs expressing the main megakaryocytic markers (**Figure 2A–B**). However, MKs differentiated with higher doses of AMG531 (1000 ng/mL and above) showed a decrease in size (**Figure 2A**) and ploidy (**Figure 2C**) that corresponded to a marked increase in MK output and proliferation (**Figure 2D**) as compared to lower AMG531 dose (100 ng/mL) and rHuTPO. Importantly, regardless of concentration, MKs differentiated in the presence of AMG531 demonstrated a defective capacity of extending proplatelets as compared to MKs cultured with rHuTPO (**Figure 2E–F**), while proplatelet morphology and tubulin distribution did not show any alteration (**Figure 2G**). Finally, in order to correlate functional outcome with biochemical signaling, we performed western blot analysis on mature MKs that demonstrated a significant increase in AKT phoshorylation in MKs cultured with higher dose of AMG531 (1000 and 2000 ng/mL) as compared to lower dose of AMG531 (100 ng/mL) and rHuTPO, while no variations were observed for pERK1/2 and pp38 (**Figure 2H**). Overall these results pointed out a different impact of increasing dose of AMG531 on MK function and biochemistry. In order to further explore this mechanism, we also asked whether increasing concentrations of rHuTPO could exert different effect on MK signalling despite the preservation of their functionality. Therefore, MKs were derived from human umbilical cord blood progenitors, as reported above, in the presence of increasing doses of rHuTPO (10 and 100 ng/mL) and western blot analysis was performed at the end of the cultures. As reported in Figure 3 we observed a significant increase in both AKT and ERK1/2 phosphorylation in MKs cultured with higher dose (100 ng/mL) as compared to lower dose (10 ng/mL) of rHuTPO. Interstingly, the observed increase in AKT and ERK1/2 phosphorylation was not paralleled by an increase of MK functionality and platelet production suggesting that a balance between AKT and ERK1/2 activation, rather than the level of phosphorylation, is necessary to sustain MK development. Importantly, in previous work [11] we showed that AKT phosphorylation occurred during proplatelet formation by human MKs; here we extend this observation by demonstrating that a modulation of AKT and ERK 1/2 phosphorylation is necessary to induce proplatelet formation, while increased levels of AKT phosphorylation lead to MK proliferation [21] with reduced ploidy and proplatelet formation.

In conclusion, our results provide novel insight into the mechanisms of MK development and proplatelet formation upon treatment with AMG531 leading to new concepts in understanding how this TPO mimetic determines and regulates MK function. Our results point out an effect of AMG531 in promoting MK proliferation rather than MK maturation and proplatelet formation (Table 2). Despite the limitation of an in vitro model, our data are important to demonstrate that increasing dose of AMG531 may exert a different effect on MK function by modulating AKT, while preserving ERK 1/2, phosphorylations.

**Table 2:** Comparative effects of rHuTPO and AMG531 on megakaryopoiesis.

	rHuTPO (10 ng/mL)	AMG531 (100 ng/mL)	AMG531 (1000 ng/mL)	AMG531 (2000 ng/mL)
**OUTPUT MK**	1,43±0,51	1,23±0,44	4,03±1,48**°** *	7,86±2,62**^#^** ^♦•^
**% PLOIDY 2N**	61,9±7,6	57,0±7,2	82,0±7,9*	79,6±5*
**% PLOIDY 4N**	18,2±4,5	22,7±1,4	14,7±5,6	15,1±2,6
**% PLOIDY 8N**	7,0±1,4	6,0±2,7	2,1±1,5	3,6±1,5
**% PLOIDY 16N**	6,6±1,5	5,7±2,6	1,2±1,1**°**	1,7±1,1**°**
**% PLOIDY 32N**	6,3±1,2	8,6±3,9	0**°** ▪	0**°** ▪
**% PROPLATELETS**	6,5±1,3	2,8±0,9 #	0,6±0,2**^#^** ^♦^	0,3±0,1**^#^** ^♦^

Values are showed as mean±SD. One-way ANOVA followed by the *post-hoc* Bonferroni’s *t*-test.

* *p*<0.05 (relative to AMG531 100 ng/mL).

**°** *p*<0.05 (relative to rHuTPO 10 ng/mL).

▪ *p*<0.01 (relative to AMG531 100 ng/mL).

# *p*<0.001 (relative to rHuTPO 10 ng/mL).

♦ *p*<0.001 (relative to AMG531 100 ng/mL).

• *p*<0.01 (relative to AMG531 1000 ng/mL).
